# Butyrate blocks cell cycle progression in colorectal cancer organoids partially through HDAC2 inhibition

**DOI:** 10.3389/fimmu.2026.1788434

**Published:** 2026-05-19

**Authors:** Banghui Liu, Yujun Huang, Xi Wang, Xiangjun Liu, Yikun Luo, Nan Wang, Hefei Tian, Lingxiao Huang, Zhenni Xu, Jinyi Lang, Xudan Lei, Dengqun Liu

**Affiliations:** 1 North Sichuan Medical College, Nanchong, China; 2 Precision Radiation in Oncology Key Laboratory of Sichuan Province, Department of Experimental Research, Sichuan Cancer Hospital & Institute, Sichuan Provincial Engineering Research Center for Tumor Organoids and Clinical Transformation, Sichuan Clinical Research Center for Cancer, Sichuan Cancer Center, School of Medicine, University of Electronic Science and Technology of China, Chengdu, China; 3 School of Basic Medical Sciences, Chengdu University of Traditional Chinese Medicine, Chengdu, China

**Keywords:** cell cycle, colorectal cancer, HDAC2, organoid, sodium butyrate

## Abstract

**Introduction:**

Butyric acid is a major gut microbiota metabolite that exhibits many biological functions, including the suppression of colorectal cancer (CRC) growth. However, previous studies have mainly been conducted using cell lines, which do not recapitulate the genuine three−dimensional spatial characteristics of CRC. In this study, we explored the therapeutic outcome and precise molecular targets of butyrate with sodium butyrate (NaB) using tumor organoids of CRC.

**Methods:**

Firstly, we examined the influence of butyrate on CRC subcutaneous allografts. And then the biological effects of butyrate were mainly determined using CRC organoids derived from KPC mice and Caco-2 cells. The morphological characteristics of butyrate-treated CRC organoids were analyzed, and multiple experimental assays were employed to determine the biological and molecular influences of butyrate. Finally, the inhibitor of HDAC2 was used to mimic the biological effects of butyrate on CRC organoids.

**Results:**

It was observed that butyrate significantly suppressed the growth of CRC allografts. Importantly, butyrate could apparently inhibit the proliferation, disrupt epithelial integrity, and induce apoptosis in CRC organoids. Transcriptomic analyses revealed that butyrate acts as an epigenetic modulator, targeting HDAC2 and selectively repressing its transcription. This led to inhibition of cyclin D1, CDK4/6, and upregulation of p21 expression, suggesting cell cycle arrest. The selective HDAC2 inhibitor similarly recapitulated the influences of butyrate on CRC organoids.

**Discussion:**

Butyrate exerts the definitive blocking effects on cell cycle progression in CRC organoids, and HDAC2 is one of the important targets. Butyrate modulates cell cycle via targeting HDAC2, constituting a novel therapeutic pathway for CRC. This study provides new evidence for gut microbial metabolites as a potential means for the prevention and treatment of colorectal cancer.

## 1 Introduction

Colorectal cancer (CRC) is the third most commonly diagnosed malignancy and the second leading cause of cancer-related death worldwide (1). Its pathogenesis involves the progressive accumulation of genomic and epigenetic alterations, leading to significant molecular heterogeneity (2). Notably, 40–45% of CRC patients harbor KRAS mutations, excluding them from anti-EGFR targeted therapies (3). Dietary factors strongly influence CRC risk: Western diets high in red meat and fat increase incidence, while fiber-rich diets are protective (4, 5). Gut microbiota links diet to cancer, and microbial dysbiosis promotes colorectal tumorigenesis (6). Butyrate, a short-chain fatty acid produced by dietary fiber fermentation, plays dual roles in intestinal homeostasis (7). Low concentrations may enhance intestinal barrier function, whereas excessive butyrate induces epithelial cell apoptosis and disrupts barrier integrity (8). It also inhibits the proliferation of stem/progenitor cells and delays wound repair (9). Nevertheless, accumulating evidence indicates that butyrate is generally protective against intestinal inflammation and carcinogenesis (10, 11).

Butyrate is a broad-spectrum inhibitor of class I histone deacetylases (HDACs), including HDAC1, HDAC2, and HDAC3 (7).HDACs refer to the family of enzymes that play an important role in chromatin remodeling and epigenetics (12). There are 18 known HDACs in humans, which are classified into four classes: Class I (HDAC1, 2, 3, 8), Class IIa (HDAC4, 5, 7, 9), Class IIb (HDAC6, 10), the NAD^+^-dependent Class III sirtuins (SIRT1-7), and Class IV (HDAC11). Except for Class III, all the other HDACs are zinc-dependent enzymes (13). Particularly, nuclear Class I isoforms (HDAC1, 2, 3, 8) regulate a wide range of critical biological processes, including cell proliferation, apoptosis, and cell cycle progression (14, 15). Histone deacetylase inhibitors (HDACis) constitute a class of potent epigenetic modulators with considerable therapeutic promise, owing to their pleiotropic actions on cellular and systemic processes (16). HDACis exert multifaceted antitumor effects through cell cycle arrest, apoptosis induction, angiogenesis modulation, and immune regulation (17).

However, most previous studies investigating the antitumor mechanisms of HDAC inhibitors have been limited to two−dimensional cell cultures (18). Here, we first used three−dimensional organoids to verify the mechanisms of butyrate and HDAC2 inhibition. Colorectal cancer organoids have significantly accelerated CRC research by faithfully recapitulating tumor pathophysiology, thus offering superior platforms for drug screening and personalized medicine compared to previous traditional 2D cell cultures (19). We established a preclinical platform of different CRC organoids derived from both murine CRC tissues and human CRC cell lines. Utilizing this system, we comprehensively assessed the therapeutic efficacy of butyrate across distinct CRC organoids and delineated the underlying molecular mechanisms. It is shown that butyrate exerts its antitumor effects partially through the transcriptional repression of HDAC2, thereby derailing cell cycle progression and compromising epithelial integrity.

## 2 Methods

### 2.1 Animals and chemicals

Adult male BALB/c mice and C57BL/6 mice (aged 6–8 weeks) were ordered from GemPharmatech (Nanjing, China). B6.Cg-*Kras^tm4Tyj^ Apc^tm1Tno^* Tg(CDX2-cre/ERT2) mice were introduced from Jackson Laboratory (Bar Harbor, ME, USA), and abbreviated as KPC mice in this study (20). All mice were housed under a specific pathogen-free (SPF) facility with controlled temperature (22 ± 1 °C), humidity (55 ± 5%), and 12-hr light/dark cycles and with *ad libitum* access to food and water. Sodium butyrate (CAS: 156-54-7, M07615) was ordered from Meryer Co. Ltd, Shanghai, and freshly prepared before experimental use. Santacruzamate A (STA, HY-N0931) was used as the specific inhibitor of HDAC2 (HDAC2i), which was the product of MedChemExpress (MCE, Shanghai, China). All experimental procedures followed the NIH Guide for the Care and Use of Laboratory Animals and were approved by the Ethics Committee of Sichuan Cancer Hospital & Institute (SCCHEC-04-2024-041).

### 2.2 Cell culture

Human colorectal cancer (CRC) cell lines Caco-2, HCT-116, and mouse CRC cell line CT-26 were obtained from the Cell Bank of Chinese Academy of Sciences (Shanghai, China) and cultured and stored in our laboratory. Cells were cultured in the required basal medium described by the supplier, which was supplemented with 10% fetal bovine serum (FBS) and 1% penicillin/streptomycin at 37 °C under 5% CO_2_ (Thermo Fisher Scientific, USA). For Caco-2 organoids, confluent monolayer cells were collected, then resuspended in Matrigel (Corning, USA). Caco-2 cells were seeded in a 96-well plate at a density of 500 cells/10 μL Matrigel to grow into organoids as previously described (21).

### 2.3 Subcutaneous CRC model

CT-26 cells were cultured and collected before use. Cells were resuspended in blank medium at a concentration of 5 × 10^6^ cells/mL. After that, cells were quickly subcutaneously injected into a BALB/c mouse. The total cell quantity was 5 × 10^5^ each mouse. Mice were then randomized into three groups: (1) Control group: normal drinking water with no supplementation; (2) Early NaB group: 100 mM NaB in drinking water and started immediately after cell inoculation; (3) Late NaB group: 100 mM NaB treatment was initiated on day 7 when tumor volumes reached ~30 mm³. Mice were allowed to take food and drink *ad libitum.* Tumor dimensions were measured every 48h using digital calipers, and the volumes of tumors were calculated (22). At day 21 after inoculation, mice were euthanized by intraperitoneal injection of an overdose of sodium pentobarbital (200 mg/kg). Tumors were collected. Their weights and images were acquired, and then the tumor tissues were fixed in 4% paraformaldehyde (PFA) for 96 hours. Tumor samples were dehydrated with xylene and gradient ethanol and embedded in paraffin. Sections were prepared for hematoxylin and eosin (H&E) and immunofluorescence (IF) staining.

### 2.4 Establishment of *in vivo* CRC model

KPC mice were used to establish an *in vivo* CRC model. Briefly, KPC mice were bred and genotyped before experimental use. The genotyping procedures were performed according to the provided protocols of Jackson Laboratory. When the offspring mice carrying target gene alleles grew up to 6~8 weeks old, they were used for the following study. Tamoxifen (Sigma Aldrich, USA) was freshly dissolved in sunflower oil at a concentration of 10 mg/mL, and then intraperitoneally injected at a dose of 2mg/20g for three consecutive days in order to induce the activation of Cre recombinase. Colon tissues were collected at day 8 after the first injection for the subsequent use of histology and organoid culture (23).

### 2.5 Colonic crypt isolation and organoid culture

KPC mice were sacrificed by cervical dislocation, and fresh colon tissues were quickly removed and flushed with ice-cold PBS. The colonic tube was longitudinally opened, then tissues were cut into 3~5 mm pieces and rinsed thoroughly with ice−cold PBS. Tissue segments were incubated in chelation buffer containing 5 mM EDTA (#25300096, Invitrogen) and 1% penicillin/streptomycin on ice for 45 min with interval agitation. After that, colonic crypts were mechanically dissociated by frequent pipetting. Supernatants containing isolated crypts were collected and centrifuged at 800 rpm for 3 minutes. Pelleted crypts were resuspended in Matrigel (#354230, Corning, USA) and seeded into 96-well flat-bottom plates. Organoids were cultured in IntestiCult™ Growth Medium (STEMCELL Technologies, Canada) supplemented with 100 μg/mL streptomycin and 100 U/mL penicillin (24). KPC organoids were treated with specific doses of NaB either at the beginning of culture or after maturation. Organoid growth was imaged by M5000 (Thermo Fisher Scientific, USA) or Cytation 5 (Agilent Technologies, USA) at 24 h, 48 h, and 72 h post-treatment. In the present study, cells or colorectal cancer (CRC) organoids were treated with NaB at a gradient concentration ranging from 0 mM to 20 mM. Based on the dose−response effects of NaB, 5 mM was selected as the working concentration for all subsequent experiments.

### 2.6 Organoid harvest and staining

KPC and Caco-2 organoids were harvested using cell recovery solution (CRS) (#354253, Corning, USA) after a 30-min incubation on ice. Briefly, after the dissociation of Matrigel, organoid suspensions were collected and gently centrifuged (800 × g, 4 °C, 3 min). Pellets were fixed in pre-chilled 4% paraformaldehyde (PFA) (#BL539A, Biosharp) for 30 min at 4 °C, then processed and embedded following our standard histological procedures. Organoid sections (4 μm thickness) were prepared and used for the following histological staining and analysis.

### 2.7 Immunohistochemical and immunofluorescent staining

Tissue or organoid paraffin slides were deparaffinized in xylene and rehydrated in gradient ethanol. Antigen retrieval was performed in Tris-EDTA buffer (#BL618A, Biosharp) at 95 °C for 20 min. Samples were blocked in PBS containing 1% BSA (#A7906, Sigma, USA) and 0.5% Triton X−100 for 1 h at room temperature. Subsequently, primary antibodies were incubated overnight at 4 °C, including anti-Ki67(Abcam, 1:400), anti-Bax (Proteintech, 1:400), anti ZO-1(Proteintech, 1:200), anti-HDAC2 (Proteintech, 1:300), anti-Cyclin D1 (Cell Signaling, 1:500), anti-p21 (Cell Signaling, 1:500), anti- CDK6 (Cell Signaling, 1:500), and anti-CDK4 (Cell Signaling, 1:500). Details of antibodies were listed in **Supplementary Table 1**. On the second day, primary antibodies were thoroughly washed away in PBS, and HRP-conjugated secondary antibody (PV-6000, ZSBio, China) was applied for 40 min at room temperature, and a DAB kit was used for immunohistochemical (IHC) based chromogen visualization (ZLI-9017, ZSBio, China). For IHC staining, nuclei were stained with hematoxylin. For immunofluorescent (IF) staining, Alexa Fluor™ 594-conjugated donkey anti-rabbit or anti-mouse IgG (A32744, A32754, Thermo Fisher, USA) was incubated for 1 h. Slides were mounted with Antifade Mounting Medium with DAPI (H-1200, Vector Labs, USA).

### 2.8 Transmission electron microscopy

Organoids were quickly collected and fixed for 1 h in cold 100 mM sodium cacodylate-HCl buffer (pH 7.4) containing 4% PFA and 1% glutaraldehyde. Tissue samples were further postfixed in 1% osmium tetroxide in 0.1 M cacodylate buffer, dehydrated in a methanol series to propylene oxide, and embedded in epoxy resin. Ultrathin sections were observed and photographed by Philips Tecnai-10.

### 2.9 Cell viability assay

Cytotoxicity of NaB on Caco-2 and CT-26 cells was determined by Cell Counting Kit-8 (CCK-8) (Dojindo, Japan). Briefly, 2×10^3^ cells per well were seeded into 96-well plates in a medium volume of 100 µL and cultured overnight. On the following day, various concentrations of NaB were added, including 0 mM,1.25 mM,2.5 mM,5 mM,10 mM, and 20mM. Cells were treated for 24 h at 37 °C. 10 µL CCK-8 reagent was added to each well and incubated in the dark for 2 h. The absorbance at 450 nm was measured using a Cytation5 microplate reader.

### 2.10 Propidium iodide staining

KPC and Caco-2 organoids in 96-well plates were treated with NaB or the HDAC2 inhibitor for 48 h. After the treatment, the culture medium was aspirated and replaced with 0.2 μg/mL PI staining solution (Beyotime, China). Organoids were stained for 20 min in PI solution. Fluorescent images were captured using an inverted M5000 microscope (Thermo Fisher Scientific, USA).

### 2.11 Organoid permeability assessment

FD4 was used to evaluate the permeability of CRC organoids. Briefly, KPC organoids were cultured and grew into mature organoids. After that, 5 mM NaB was loaded into the medium to treat organoids for 48 h. The Organoid barrier integrity was evaluated by FITC-labeled Dextran (MW = 4000) (FD4). Organoids were incubated with 3 mg/mL FD4 at 37 °C for 3 h in the dark. After the incubation, free FD4 in the medium was completely washed out, and fluorescent images of organoids stained by FD4 were captured by Cytation 5.

### 2.12 RNA isolation and qRT-PCR

Total RNA from KPC or Caco-2-derived organoids was extracted by RNAiso Plus (Takara, Japan). RNA quality and concentration were determined by Nanodrop 2000. cDNA synthesis was performed using Hifair II 1st Strand cDNA Synthesis Super Mix (YEASEN, China). qPCR reactions were conducted by Hifair qPCR SYBR Green Master Mix (YEASEN, China) on a Bio-Rad CFX96 system. Primer sequences used in this study are listed in **Supplementary Table 2**. Amplification of target genes was performed on Bio-Rad iQ5. β-Act*in* was used as a loading reference to normalize gene expression. mRNA expression was calculated via the 2*^−ΔΔCt^* method.

### 2.13 RNA-seq assay

Total RNA from control and NaB-treated organoids was isolated using RNAiso Plus and used for transcriptome sequencing following our previous work (25). Briefly, RNA integrity assessment, library construction, and Illumina sequencing were performed under the standardized protocols. Bioinformatics analysis was performed by OE Biotech, Shanghai. Differentially expressed genes (DEGs) were calculated as fold change > 1.5 or < 0.5. Functional enrichment analysis included KEGG pathways and Wikipathways, MetaboAnalyst, Gene Set Enrichment Analysis (GSEA), etc. Key DEGs were also validated by qRT-PCR assay.

### 2.14 Statistical analysis

All the data were presented as the mean ± SD, and analyzed using GraphPad Prism 10 (GraphPad Software, USA). Comparison between two different groups was conducted by a two-tailed unpaired Student’s *t*-test or a non-parametric test, depending on whether the data conformed to normal distribution. Comparison of multiple groups was analyzed using one-way ANOVA with *post-hoc* Tukey’s test. *P* values less than 0.05 were considered statistically significant. The different symbols of statistical results were indicated by *: *P* < 0.05, **: *P* < 0.01, ***: *P* < 0.001, ****: *P* < 0.0001. *P >*0.05 was considered as not significant (n.s).

## 3 Results

### 3.1 Butyrate inhibits the growth of CRC subcutaneous allografts

**Figure 1:**
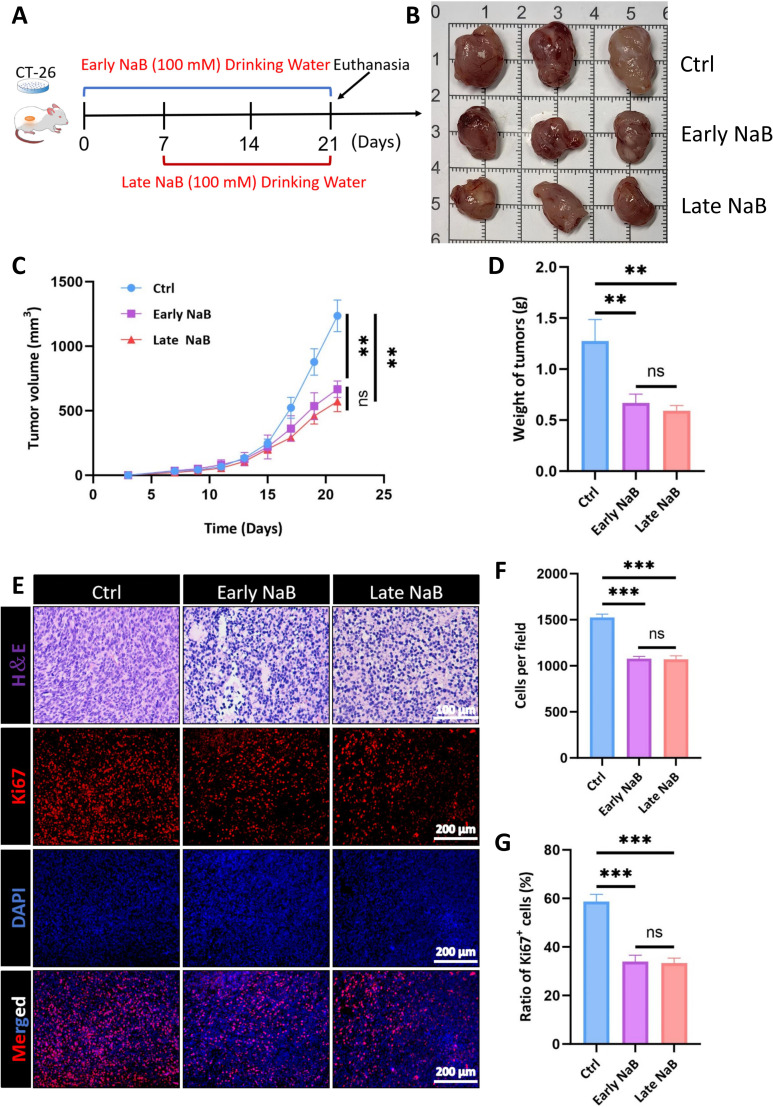
Butyrate suppresses tumor growth and proliferation in CRC allografts. **(A)** Schematic cartoon of CT-26 subcutaneous allografts and different NaB treatment strategies. **(B)** The gross images of CT-26 derived CRC tumors in each group (n=3). **(C)** The growth curve of tumor volumes in the three groups at different times. **(D)** The average weight of tumors in NaB treated mice was significantly decreased compared to the control group (n=6). **(E)** Hematoxylin & eosin (H&E) (Bar=100µm) and Ki67 fluorescent staining of CRC tissues in the control group, Early NaB group, and Late NaB group (Bar=200µm). **(F)** Statistical analysis for the number of cells in the observed images (n=3). **(G)** Quantitative results of Ki67+ positive cells in the tumor tissues with or without NaB treatment (n=3). **P < 0.01, ***P < 0.001, n.s, not significant.

To verify that butyrate suppresses the growth of colorectal cancer cells in murine models, previous studies have established xenograft tumors by injecting HCT116, SW480, and Hep3B cells into the right flank of 6- to 8-week-old BALB/c nude mice (26). At the beginning of this study, we first evaluated whether NaB was able to inhibit the growth of CRC *in vivo*. Mouse derived CT-26 cells were subcutaneously inoculated in immunocompetent BALB/c mice to establish the CRC allograft model. NaB (100 mM) was administered via drinking water at different stages of tumor growth to determine its effects on the growth of CRC allografts (**Figure 1A**). NaB treatment significantly inhibited tumor growth, as reflected by the pronounced decrease in both tumor weight and volume as compared with the vehicle-treated control group (**Figures 1B–D**). Interestingly, the initial time of NaB administration did not yield statistically divergent outcomes in terms of tumor mass or growth metrics, indicating a robust efficacy of NaB independent of intervention strategies. Moreover, we also examined the histopathological characteristics of tumor tissues in different groups. Hematoxylin & eosin (H&E) staining showed more active tumor growth in the control group, and both early and late NaB groups had less cellular content compared with mice without NaB treatment (**Figures 1E, F**). These results were identical to the decreased tumor volumes. In addition, we also employed immunofluorescent (IF) staining against Ki67 to examine the proliferation level, and there were significantly fewer Ki67+ proliferative cells within NaB-treated CRC tumor tissues (**Figures 1E, G**). Therefore, these data demonstrate that butyrate is capable of inhibiting the growth and proliferation of CRC allograft *in vivo*.

### 3.2 Butyrate blocks the expansion of different CRC organoids

**Figure 2:**
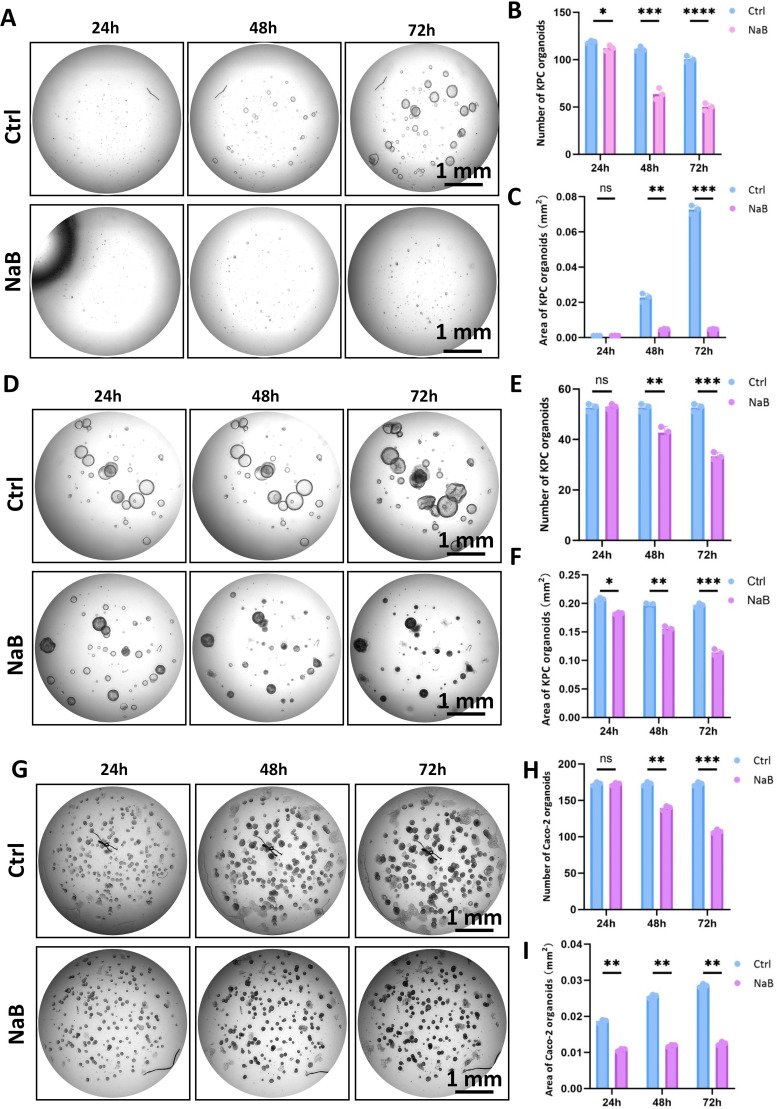
Butyrate inhibits the growth of CRC organoids derived from KPC mice and the Caco-2 line. **(A)** NaB treatment (0.5 mM) at the initial seeding stage blocked the formation of KPC organoids compared to the Ctrl group (KPC organoids without the loading of NaB) (Bar=1mm). **(B)** Quantification of the number of KPC organoids at different times after NaB treatment. **(C)** Statistical analysis for the area of KPC organoids after the administration of NaB. **(D)** NaB treatment (5 mM) at the mature stage also caused breakdown of KPC organoids in comparison with the Ctrl group (KPC organoids without the loading of NaB) (Bar=1mm). **(E)** Statistical analysis for the number of mature KPC organoids at different times after NaB treatment. **(F)** Quantification of the area of mature KPC organoids after NaB treatment. **(G)** NaB treatment (5 mM) initiated after maturation of Caco-2 organoids suppressed their growth (Bar=1mm). **(H)** Statistics for the number of mature Caco-2 organoids after NaB treatment. **(I)** Quantification for the growing area of Caco-2 organoids in the presence of NaB. *P < 0.05, **P < 0.01, ***P < 0.001, ****P < 0.0001.

In the previous section of this study, we found that butyrate could significantly suppress the growth of subcutaneous CRC allografts in mice. However, it is known that butyrate has multiple biological functions, and the *in vivo* contexts present a comprehensive outcome, including tumor cells, immune cells, fibroblasts, etc. We aimed to explore the direct biofunctions of butyrate on CRC cells. Since tumor organoids can better present the three-dimensional characteristics of cancer cells than traditional 2D cultures, we established two colorectal cancer organoids from transgenic CRC mice and Caco-2 cells. Mice carrying the KRAS^G12D^ mutation and with conditional knockout of the APC gene were introduced from Jackson Laboratory. After breeding, KPC mice with an ideal genotype were used to establish a CRC model by intraperitoneal injection of 2mg/20g tamoxifen (**Supplementary Figure 1A**). The cecum and colon of KPC mice became thicker than those of healthy control mice after tamoxifen treatment, as indicated by black arrows (**Supplementary Figure 1B**), especially in the proximal colon (**Supplementary Figure 1B**). H&E staining revealed the disorganized colonic epithelial architecture and malignant transformation and confirmed the successful establishment of the CRC model (**Supplementary Figure 1C**). Then we isolated colonic crypts from KPC mice to culture CRC organoids and test the influences of NaB on these organoids. KPC organoids were treated by NaB in two different strategies, including immediate treatment after organoid seeding (**Figure 2A**; **Supplementary Figure 1D**) and mature treatment after 4-day culture (**Figure 2D**; **Supplementary Figure 1G**). The parameters of organoids, including number and area, were quantified. The results showed that NaB treatment significantly inhibited the growth of KPC organoids, regardless of whether it was applied immediately after seeding or post-maturation (**Figures 2B, C, E, F**; **Supplementary Figures 1E, F, H, I**). Meanwhile, human CRC organoids derived from Caco-2 cells were also similarly sensitive to NaB treatment (**Figure 2G**; **Supplementary Figures 2A, D**). NaB greatly decreased the growth of Caco-2 organoids in a time- and dose-dependent manner (**Figures 2H, I**; **Supplementary Figures 2B, C, E, F**). We also cultured HCT-116 cells in Matrigel and found NaB could inhibit the growth of tumor spheres (**Supplementary Figure 3**). Therefore, these results demonstrate that butyrate could suppress the growth and expansion of CRC organoids *in vitro*.

### 3.3 Butyrate decreases the proliferation of CRC organoids and induces apoptosis

**Figure 3:**
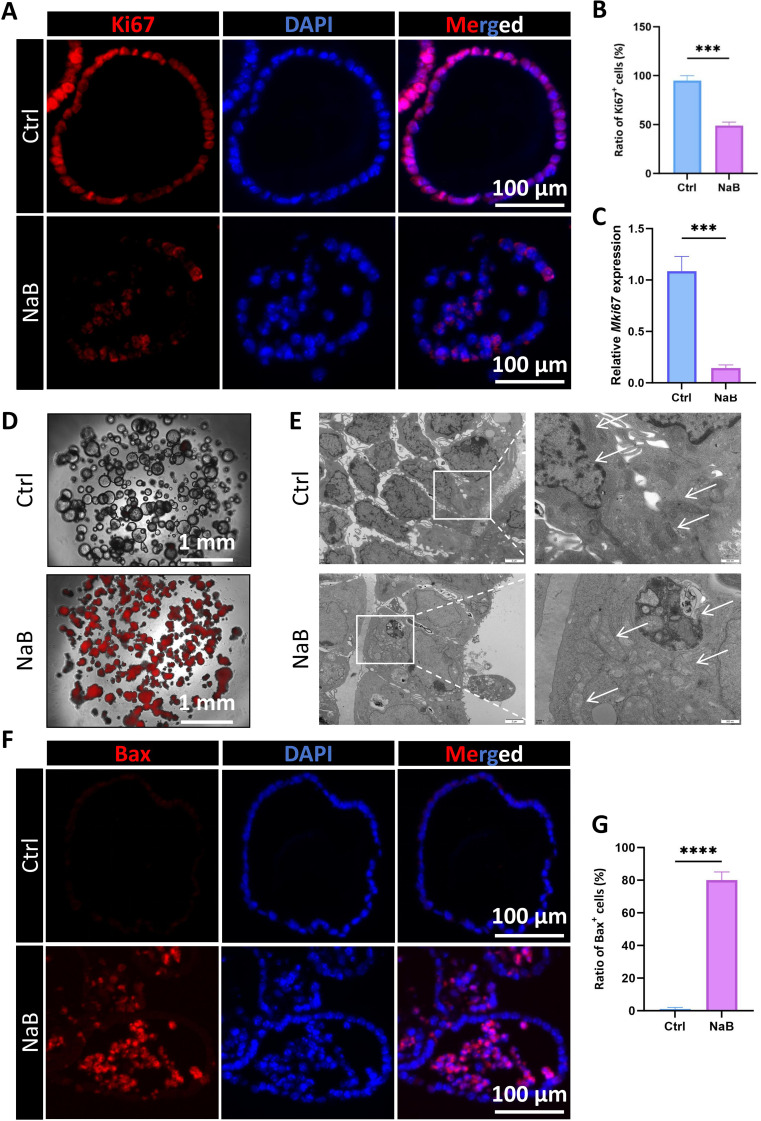
Butyrate suppresses cell proliferation and induces apoptosis in KPC organoids. **(A)** Representative immunofluorescent images of Ki67 in the control group and NaB-treated KPC organoids (Bar=100µm). **(B)** Quantification for the ratio of Ki67^+^ epithelial cells per KPC organoids. **(C)** qRT-PCR analysis for the relative mRNA level of *Mki67* gene in KPC organoids. **(D)** The administration of NaB significantly increased organoid cell death of the KPC organoid, which was shown by PI staining. **(E)** NaB treatment caused cell shrinkage, chromatin condensation, autophagosome formation, and mitochondrial swelling with cristae disruption, indicating apoptotic cell death (High magnification scale bar = 500 nm; low magnification scale bar = 2 μm). **(F)** Representative immunofluorescent images of Bax in the control group and NaB-treated KPC organoids (Bar=100µm). **(G)** Quantification for the ratio of Bax^+^ epithelial cells per KPC organoids. *P < 0.05, ***P < 0.001, ****P < 0.0001.

To further validate the suppressing effects of butyrate, we employed a comprehensive analysis using KPC and Caco-2 organoids. Organoids were processed for histological evaluation, including fixation, sectioning, and immunofluorescence staining. Here, we treated these two organoids with NaB for 48 hours. And the staining results demonstrated that NaB treatment led to a significant decrease of Ki67^+^ cells in both KPC and Caco-2 organoids as compared to the control group (**Figures 3A, B**; **Supplementary Figures 4A, B**). The qRT-PCR results identified that butyrate significantly decreased the mRNA level of the *Mki67* gene in KPC and Caco-2 organoids (**Figure 3C**; **Supplementary Figure 4C**). In addition to the inhibition on cell proliferation, propidium iodide (PI) staining also revealed an increase in the mortality of organoid upon NaB exposure (**Figure 3D**; **Supplementary Figure 4D**), suggesting that butyrate could enhance cell death in CRC organoids. Transmission electron microscopy (TEM) revealed that control cells exhibited intact morphology, continuous cell membranes, evenly distributed cytoplasm, regularly organized organelles, and normal mitochondria with densely packed, well−arranged cristae, with no signs of cell damage or death. After NaB treatment, cell density and number were significantly reduced, and autophagosomes were observed. Cells exhibited shrunken morphology, impaired membrane integrity, and chromatin condensation and margination, typical of apoptosis. Meanwhile, mitochondria were markedly swollen, with disrupted cristae, disorganized arrangement, and matrix cavitation, indicating severe mitochondrial damage (**Figure 3E**). BCL-2-associated X protein (Bax) is a core pro-apoptotic effector protein of the Bcl-2 family and is primarily responsible for mediating mitochondrial outer membrane permeabilization (MOMP), representing a key rate-limiting step in the intrinsic apoptotic pathway, so we stained Bax using butyrate-treated KPC organoids. The immunohistochemical staining of Bax demonstrated that butyrate treatment led to a significant increase in Bax^+^ cells in KPC organoids as compared to the control group (**Figures 3F, G**). Moreover, we validated these results in conventional 2D cultured human Caco-2 and murine CT-26 cell lines. With the increasing of NaB doses, both Caco-2 cells and CT-26 cells exhibited an apparently decreased cell viability (**Supplementary Figure 4E**). Hence, these data support that butyrate was able to inhibit the cell proliferation and induce apoptosis of CRC organoids.

### 3.4 Butyrate breaks epithelial integrity and increases the permeability of CRC organoids

**Figure 4:**
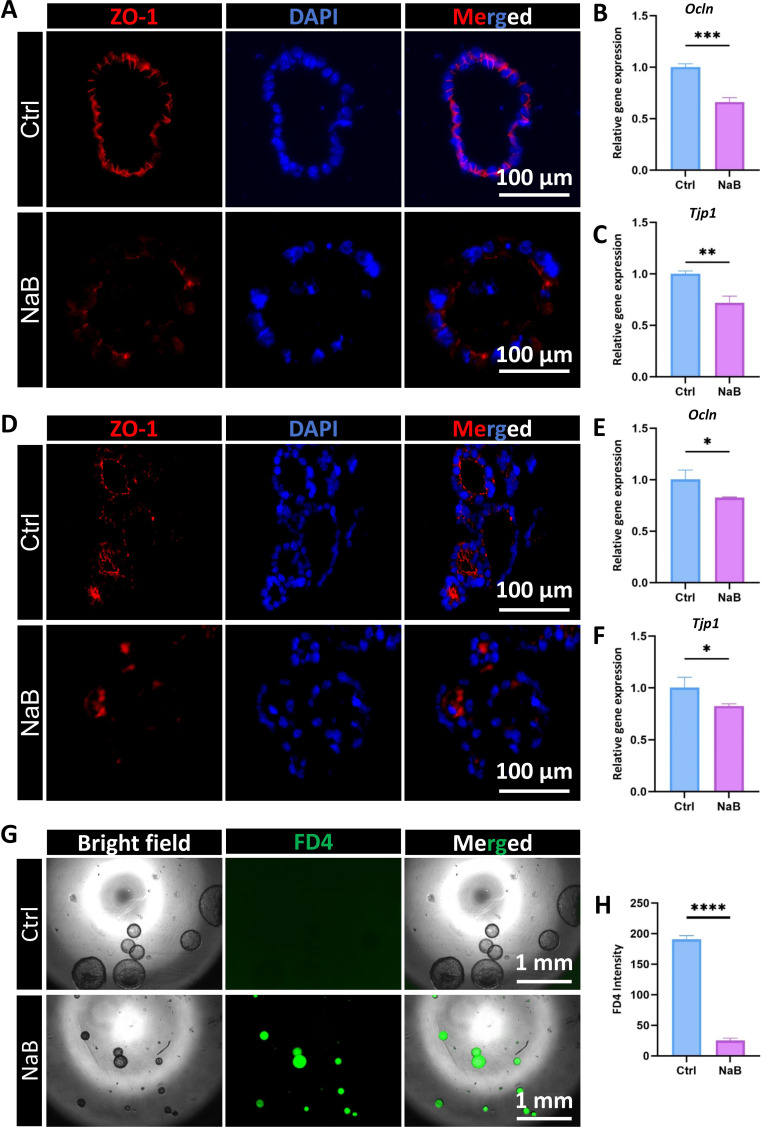
Butyrate interrupts epithelial tight junction and increases paracellular permeability in CRC organoids. **(A)** Representative immunofluorescent images of ZO-1 between control and NaB-treated KPC organoids (Bar=100µm). **(B)** qRT-PCR results for the mRNA level of the *Ocln* gene in these two KPC organoid groups. **(C)** The qRT-PCR assay showed significantly less mRNA expression of the *Tjp1* gene. **(D)** Representative IF images of ZO-1 staining in control and NaB-treated Caco-2 organoids (Bar=100µm). **(E)** Relative gene expression level of *Ocln* in Caco-2 organoids with or without NaB treatment. **(F)** qRT-PCR results showed a decreased mRNA level of the *Tjp1* gene after NaB treatment in Caco-2 organoids. **(G)** NaB apparently increased the leakage of FD4 dye into the lumen of KPC organoids (Bar=1mm). **(H)** Quantification results for the fluorescent intensity of FD4 dye in the lumen of KPC organoids. *P < 0.05, **P < 0.01, ***P < 0.001, ****P < 0.0001.

Given the central role of tight junctions in maintaining epithelial barrier integrity and cellular polarity, their dysregulation is critically implicated after many cancer treatments. In this study, we also examined the biological impairments of butyrate on the integrity and permeability of CRC organoids. We passaged KPC organoid pieces and Caco-2 cells into Matrigel and cultured them to grow into mature organoids, then we treated these different CRC organoids with 5mM NaB for 48 hours. Both organoid tissue samples and RNA samples were collected for subsequent analysis. Immunofluorescent (IF) staining identified that NaB caused a significant reduction of ZO-1 signals (**Figure 4A**). Consistent with the reduced protein staining, qRT-qPCR analysis also demonstrated a significantly downregulated mRNA expression level of *Ocln* and *Tjp1*in KPC organoids after NaB treatment compared to the control group (**Figures 4B, C**). The similar trends happened in NaB-treated Caco-2 organoids (**Figures 4D, E, F**). While ZO-1 staining and tight junction related gene expressions (*Ocln* and *Tjp1*) were reduced after NaB treatment, we also examined the epithelial permeability of KPC organoids by FITC-labeled dextran 4000 (FD4). To functionally assess whether these molecular changes could definitely harm the epithelial permeability, both control and NaB-treated KPC organoids were incubated with FD4. After appropriate incubation, fluorescence images clearly showed a pronounced FD4 leakage into the lumen of NaB-treated organoids, whereas controls exhibited no FD4 dye penetration (**Figures 4G, H**). This elevated FD4 permeability not only corroborated the molecular findings but also underscored a potential mechanism by which butyrate may compromise the barrier function of CRC organoids, ultimately inhibiting cancer progression and metastatic capacity. So, these results provide experimental evidence that butyrate could disrupt the epithelial integrity and permeability of CRC organoids.

### 3.5 Butyrate suppresses cell cycle-related proteins and HDAC2 expression

**Figure 5:**
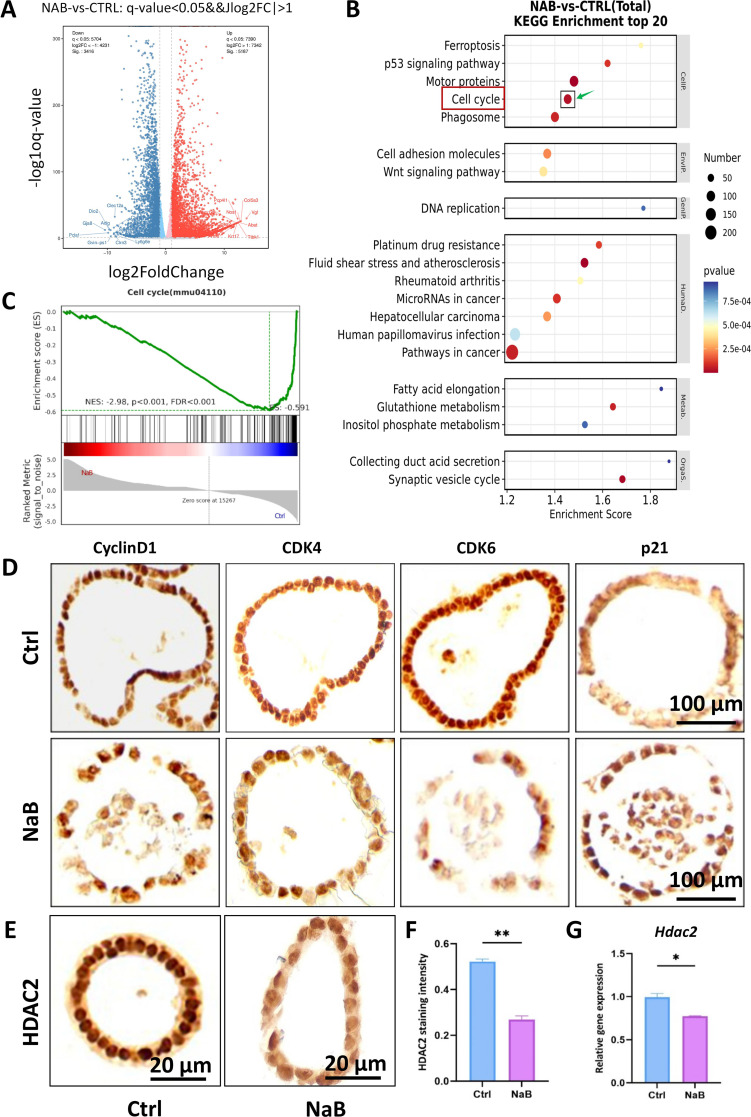
Butyrate suppresses cell cycle-related proteins and HDAC2 expression in KPC organoids. **(A)** Volcano plot of DEGs between two groups of KPC organoids. **(B)** KEGG pathway analysis highlighted the Cell Cycle as a key enrichment after NaB treatment. **(C)** GSEA plots confirm a significant downregulation of hallmark genes in DNA replication. **(D)** IHC validation for the protein levels of Cyclin D1, CDK4, CDK6, and p21 in control and NaB-treated KPC organoids (Bar=100µm). **(E)** IHC staining of HDAC2 revealed the decreased protein levels of HDAC2 in the nucleus of KPC organoids (Bar=20µm). **(F)** Statistical analysis for the staining intensity of HDAC2 in the two groups. **(G)** qRT-PCR validation for the gene expression level of *Hdac2* in KPC organoids after the administration of NaB *P < 0.05, **P < 0.01, ***P < 0.001, ****P < 0.0001.

To comprehensively delineate the transcriptional landscape caused by NaB, we performed RNA sequencing using both NaB-treated and control KPC organoids. Principal component analysis (PCA) revealed a clear separation of these two groups, while replicates within each group clustered tightly, affirming high experimental reproducibility (**Supplementary Figure 5A**). Comparative analysis identified a total of 8,603 differentially expressed genes (DEGs), comprising 5,187 upregulated and 3,416 downregulated transcripts in response to NaB treatment (**Supplementary Figure 5B**). A volcano plot further underscored the extensive nature of these transcriptional alterations, visualizing a substantial number of statistically significant DEGs (**Figure 5A**). KEGG enrichment analysis revealed that the “cell cycle” pathway was the most significantly downregulated among all DEGs (**Figure 5B**; **Supplementary Figures 5C, D**). Gene Set Enrichment Analysis (GSEA) corroborated these targeted results, predominantly downregulated upon NaB exposure (**Figure 5C**). Reinforcing this finding, WikiPathways analysis specifically highlighted a marked enrichment for genes governing the transition from G1 phase to S phase (**Supplementary Figures 5E, F**). Analysis of FPKM values showed a coherent expression pattern and indicated genes encoding key positive regulators of the cell cycle, including Cyclin D1 (*Ccnd1*), *Cdk4*, and *Cdk6*, were downregulated, whereas those encoding cyclin-dependent kinase inhibitors (eg. *Cdkn1a*) were upregulated (**Supplementary Figure 5G**). This transcriptional profile was corroborated at the protein level by immunohistochemical (IHC) staining, which showed concordant alterations in the abundance of the corresponding proteins, including Cyclin D1, CDK4, CDK6, and p21 (**Figure 5D**). Given the established role of butyrate as a histone deacetylase inhibitor (HDACi), we next examined its impact on the expression of HDACs. Our transcriptomic data showed that NaB triggered a complex regulatory response. The upregulated HDAC genes included *Hdac1*, *Hdac3*, *Hdac5*, and *Hdac6*, while those downregulated HDAC genes contained *Hdac2*, *Hdac4*, and *Hdac7-11* (**Supplementary Figure 6A**). Among the members of class I HDACs, *Hdac2* and *Hdac8* are significantly downregulated. Since the background expression level of *Hdac8* is much lower than that of *Hdac2*, we subsequently focused on *Hdac2*. IHC analysis confirmed a marked reduction of HDAC2 protein in NaB-treated organoids compared to controls (**Figures 5E, F**). Subsequent qRT-PCR quantification demonstrated that within class I HDAC family members, *Hdac2* expression was significantly decreased, while *Hdac1* and *Hdac3* were markedly increased (**Figure 5G**, **Supplementary Figures 6B, C**). Therefore, we propose a model wherein the tumor-suppressive effect of butyrate is mediated, at least in part, through the transcriptional suppression of HDAC2.

### 3.6 HDAC2 inhibitor recapitulates the influences of butyrate on CRC organoids

**Figure 6:**
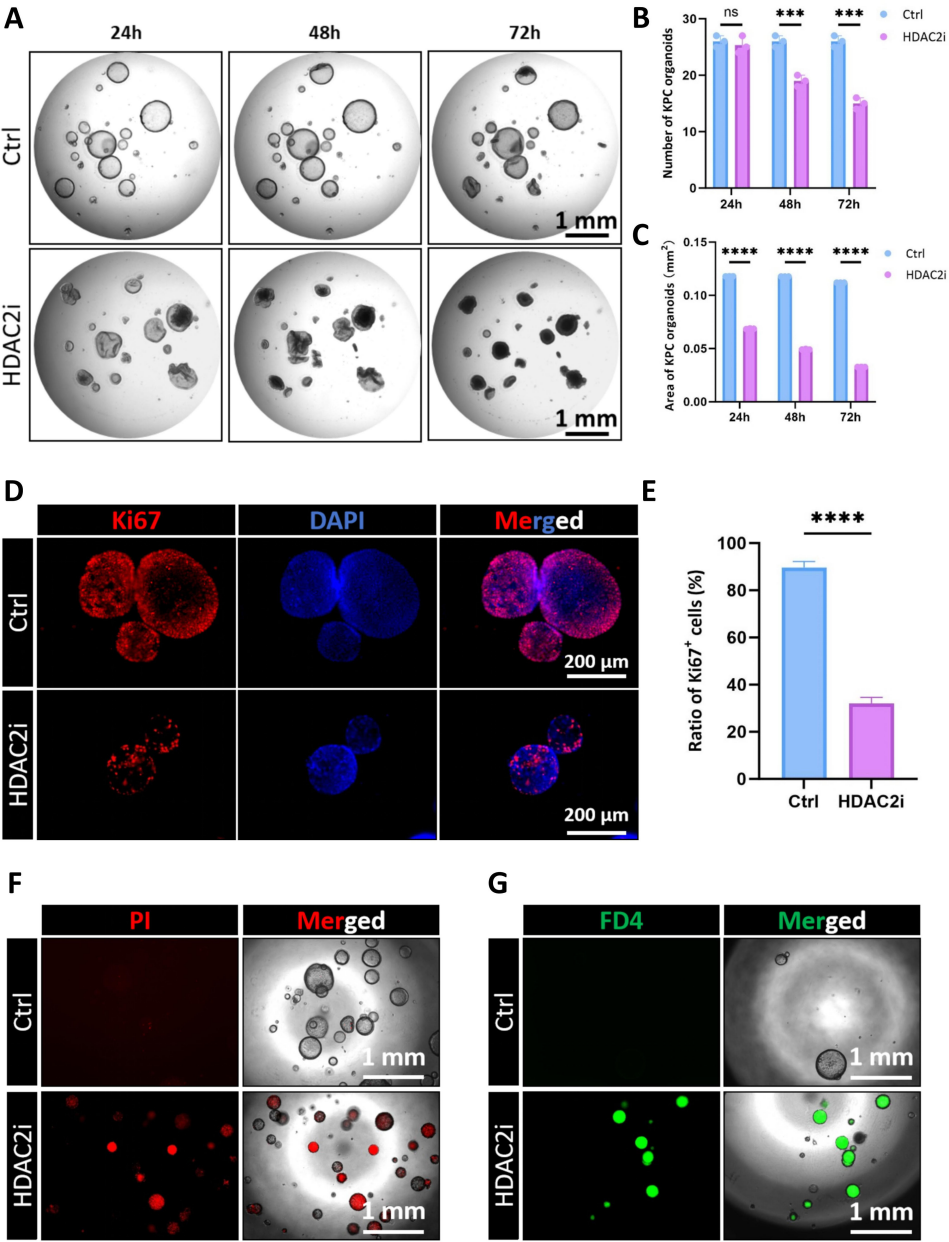
Pharmacological inhibition of HDAC2 recapitulates the biological influences of butyrate on CRC organoids. **(A)** HDAC2 inhibitor (HDAC2i) significantly inhibited the growth of KPC organoids in a time- and dose-dependent manner (Bar=1mm). **(B)** Quantification analysis for the number of KPC organoids in the presence of HDAC2i. **(C)** Quantitative results for the area of KPC organoids with or without HDAC2i treatment. **(D)** Representative IF images of Ki67 staining in KPC organoids after HDAC2i treatment (Bar=200µm). **(E)** Quantitative analysis for the ratio of Ki67+ cancer cells in KPC organoids in the presence of HDAC2i. **(F)** HDAC2i increased the amount of PI-positive KPC organoids (Bar=1mm). **(G)** HDAC2i enhanced the organoid permeability shown by the FD4 assay. ***P < 0.001, ****P < 0.0001. ns, not significant.

To decisively validate our hypothesis that HDAC2 inhibition is the principal mechanism underlying the antitumor activity in CRC organoids, we employed a selective HDAC2 inhibitor (HDAC2i) to treat KPC organoids. Strikingly, the administration of HDAC2i recapitulated the phenotypes of NaB, inhibiting organoid cell proliferation in a clear time- and dose-dependent manner (**Figures 6A–C**, **Supplementary Figure 7**). The anti-proliferative effect of HDAC2i was also confirmed by *in situ* Ki67 staining in KPC organoids (**Figures 6D, E**), which revealed a significant reduction in the proliferation marker within HDAC2i-treated organoids compared to their control counterparts. Beyond impairing cell proliferation, HDAC2 inhibition also induced cell death and disrupted epithelial integrity as evidenced by significantly increased fluorescence intensity in both propidium iodide (PI) and FD4 permeability assays (**Figures 6F, G**). The increased PI staining indicated a higher rate of cell death, while the enhanced FD4 penetration demonstrated a loss of barrier function of CRC organoids due to compromised tight junctions. The concordance of these findings, including suppressed cell proliferation, increased cell death, and enhanced permeability, closely mirrored the comprehensive phenotypic profiles observed after NaB treatment. Therefore, these experiments strongly suggest that the targeted blocking of HDAC2 signaling could recapitulate the biological inhibition of butyrate on CRC organoids.

## 4 Discussion

The intricate interplay between gut microbiota, their metabolites, and host epithelial cells constitutes a critical axis in colorectal cancer (CRC) pathogenesis and therapeutic outcomes (27). Among the variety of microbial metabolites, butyrate has emerged as a key mediator with a definitive antitumor activity, although its underlying mechanisms have not been fully clarified (28). However, many prior *in vitro* studies have been performed mainly depending on butyrate’s efficacy across multiple CRC cell lines (e.g., SW480, LOVO, HCT116, HCT8) (29), revealing pleiotropic biological effects. For example, butyrate could inhibit cell migration via miR-200c-mediated downregulation of Bmi-1 (30) and suppress angiogenesis and metastasis via interference with the Sp1/neuropilin-1/VEGF axis (31). Butyrate could also impair cell proliferation and colony formation via ERK2/MAPK-dependent endocan secretion (32) and induce apoptosis via Wnt/β-catenin pathway suppression (33). In addition, butyrate is also capable of reinforcing antitumor immunity by modulating CD8^+^ T cells (34). Unfortunately, more and more scientists realize that traditional cell lines cannot mimic the biological and spatial characteristics of *in vivo* cancers. Therefore, in this study, we aimed to explore butyrate’s activity by dissecting its role as a precision epigenetic regulator in the model of CRC organoids.

The therapeutic targeting of CRC carrying KRAS mutation represents a persistent clinical challenge, because KRAS and other mutations occur in 40–45% of cases and confer resistance to anti-EGFR therapies (35). These mutations independently correlate with increased metastatic potential and poor prognosis (36), and they are frequently associated with an immune-excluded tumor microenvironment that predicts poor response to immunotherapy (37, 38). To address this unmet need, we employed KRAS-mutated organoids as physiologically relevant ex vivo models that faithfully retain the spatial and molecular characteristics of original CRCs (39, 40). These 3D culture systems have emerged as powerful platforms for dissecting cancer heterogeneity and tumor-microenvironment interactions (41), which provides an ideal context for evaluating butyrate’s mechanism of action in molecularly defined CRC subtypes.

In the current study, we first confirmed that butyrate significantly suppressed the growth of CRC allografts *in vivo*. However, the tumor microenvironment of CRC is composed of different cell components, and butyrate is capable of regulating both tumor cells and immune cells. Here, we mainly want to investigate its functions on the cancer cells of CRC. Subsequently, we demonstrated that butyrate potently inhibited cell proliferation and induced apoptosis in multiple organoid models. Identifying the specific molecular target of butyrate is critical for understanding its mechanism of action. While previous studies have often described butyrate as a pan-HDACi, our transcriptomic profiling revealed a more nuanced and selective effect. Among the diverse HDAC family members, HDAC2 was the most significantly downregulated class I HDAC. Frequently overexpressed in CRC, the class I histone deacetylase HDAC2 is a key driver of cell cycle progression and oncogenic transformation (42). HDAC2 is also implicated as an independent prognostic factor for adverse outcomes in diverse cancers, including those of the oral cavity cancer, prostate cancer, and gastric cancer (43). Our data unequivocally established the pivotal link between butyrate and HDAC2, and we found that a selective HDAC2 inhibitor (HDAC2i) could recapitulate the phenotypic hallmarks of NaB on CRC organoids, including compromised epithelial integrity, suppressed proliferation, and enhanced cell death. This functional convergence strongly supports that the inhibition effects of butyrate are mediated partially through HDAC2 suppression. Some studies have shown that butyrate could serve as a potent endogenous inhibitor of classical Zn²^+^-dependent class I, II, and IV histone deacetylases (HDACs), exhibiting a broad-spectrum biological activity (16, 44, 45). HDAC inhibitors (HDACis) exert pleiotropic effects on cell cycle progression, differentiation, and cell death modalities (apoptosis, necrosis, autophagy) (16). These results are consistent with our observations.

These findings establish a coherent link from microbial ecology to epigenetically guided precision medicine. We demonstrate that the commensal metabolite butyrate functions as a targeted epigenetic modulator, exerting transcriptional repression of HDAC2. This inhibition disrupts colorectal cancer progression through two parallel mechanisms: impeding cell cycle progression and weakening epithelial integrity. Thus, butyrate is repositioned from a nutritional derivative to a promising therapeutic agent. Notably, butyrate exhibited broad activity even in KPC organoids carrying Kras mutations. The susceptibility of these resistant models to butyrate implies that its epigenetic mode of action may circumvent traditional signaling pathways disabled by KRAS mutations, revealing a potential treatment strategy for this clinically challenging patient subgroup.

We have unequivocally demonstrated that the inhibition effects of butyrate are mediated partially through HDAC2 suppression. This primary molecular event serves as the critical trigger for a cascade of antitumor responses, including the profound disruption of the cell cycle, evidenced by the suppression of Cyclin D1/CDK4/6 and induction of p21, and the significant compromise of epithelial barrier integrity through the loss of tight junction proteins such as ZO-1. The striking recapitulation of butyrate’s phenotypic spectrum, from cell cycle arrest and apoptosis to increased permeability, upon specific HDAC2 inhibition (HDAC2i), provides compelling functional validation of HDAC2 as the keystone target. Furthermore, the potent efficacy of butyrate in aggressive, KRAS-mutant organoids highlights its potential to address a major clinical challenge in CRC therapy. However, our study has not performed mechanistic validation using cell models with HDAC2 knockdown or knockout, which represents a limitation of the present work. Further in-depth investigations are warranted in the future to clarify the precise molecular targets and regulatory pathways involved.

## 5 Conclusion

In summary, our study delineates a coherent and mechanistically grounded pathway through which the gut microbial metabolite butyrate exerts its antitumor effects in colorectal cancer. It bridges the gap between a common microbial metabolite and a targeted epigenetic mechanism, and our work repositions sodium butyrate from a dietary-derived compound into a promising therapeutic candidate. It firmly identifies butyrate-mediated HDAC2 suppression in cell cycle control as a scientifically validated and therapeutically relevant pathway, offering a novel strategic direction for the epigenetic management and intervention of CRC.

## Data Availability

The raw data supporting the conclusions of this article will be made available by the authors, without undue reservation.
