# Supplementary material for: Butyrate blocks cell cycle progression in colorectal cancer organoids partially through HDAC2 inhibition
Source: Front Immunol. 2026 May 19;17:1788434. doi: 10.3389/fimmu.2026.1788434 (PMC13226563; doi:10.3389/fimmu.2026.1788434)
Supplement: Supplementary file 1 [file Table1.docx]

**
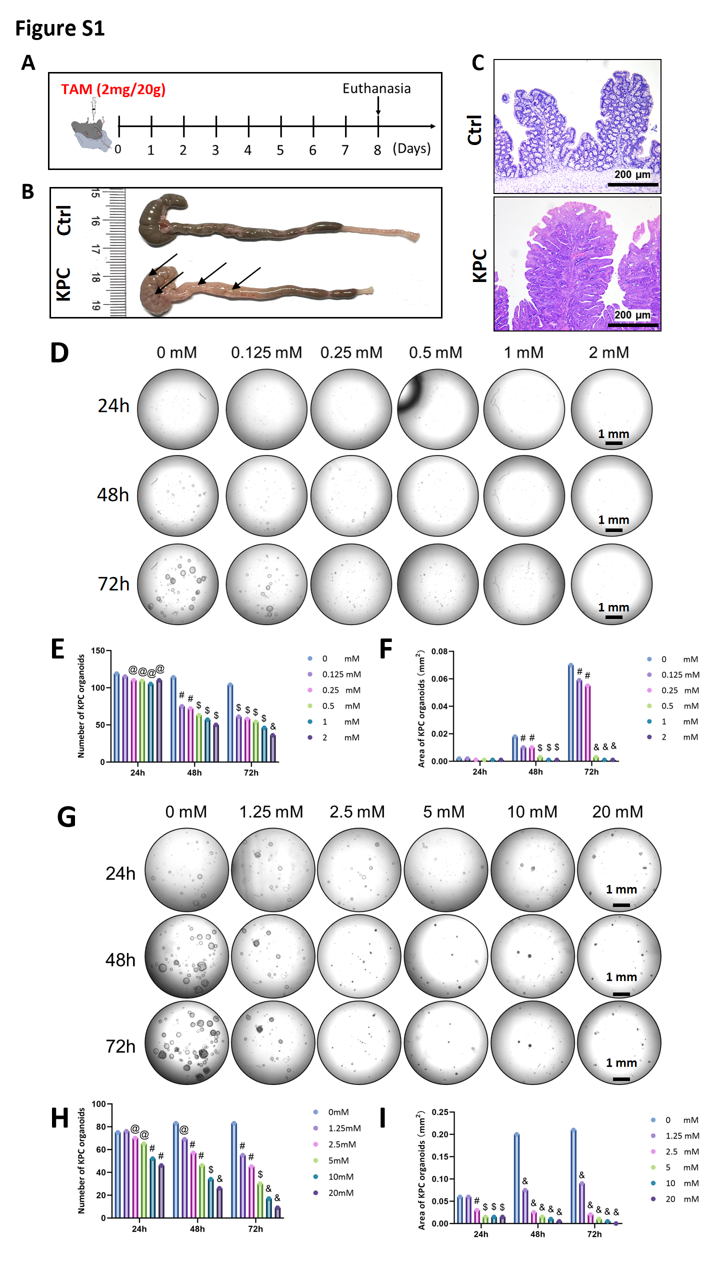
**

**Figure S1.** **Butyrate dose-dependently inhibits the growth of KPC organoids. (A)** Schematic diagram demonstrated the treatment of control C57BL/6 mice and KPC mice by tamoxifen. **(B)** The gross images of cecum and colon in healthy control mouse and KPC mouse. **(C)** Representative histological images of normal colonic epithelium and colorectal cancer tissue after tamoxifen treatment (Bar=200µm). **(D)** KPC organoids were treated by NaB with a range from 0.125 mM to 2 mM immediately started after seeding into Matrigel (Bar=1mm). **(E)** Quantification for the number of KPC organoids at the early stage of growth. **(F)** Statistical analysis for the area of KPC organoids treated by NaB at the early stage of growth. **(G)** Given after the mature of KPC organoids, NaB treatment ranging from 1.25 mM to 20 mM exhibited a significant inhibition on the organoid growth (Bar=1mm). **(H)** Quantification for the number of mature KPC organoids at different times of NaB treatment. **(I)** Quantitative analysis for the area of mature KPC organoids after NaB treatment. @: *: *P*<0.05, #: **: *P*<0.01, $: ***: *P*<0.001, &: ****: P <0.0001; n.s: not significant.

**
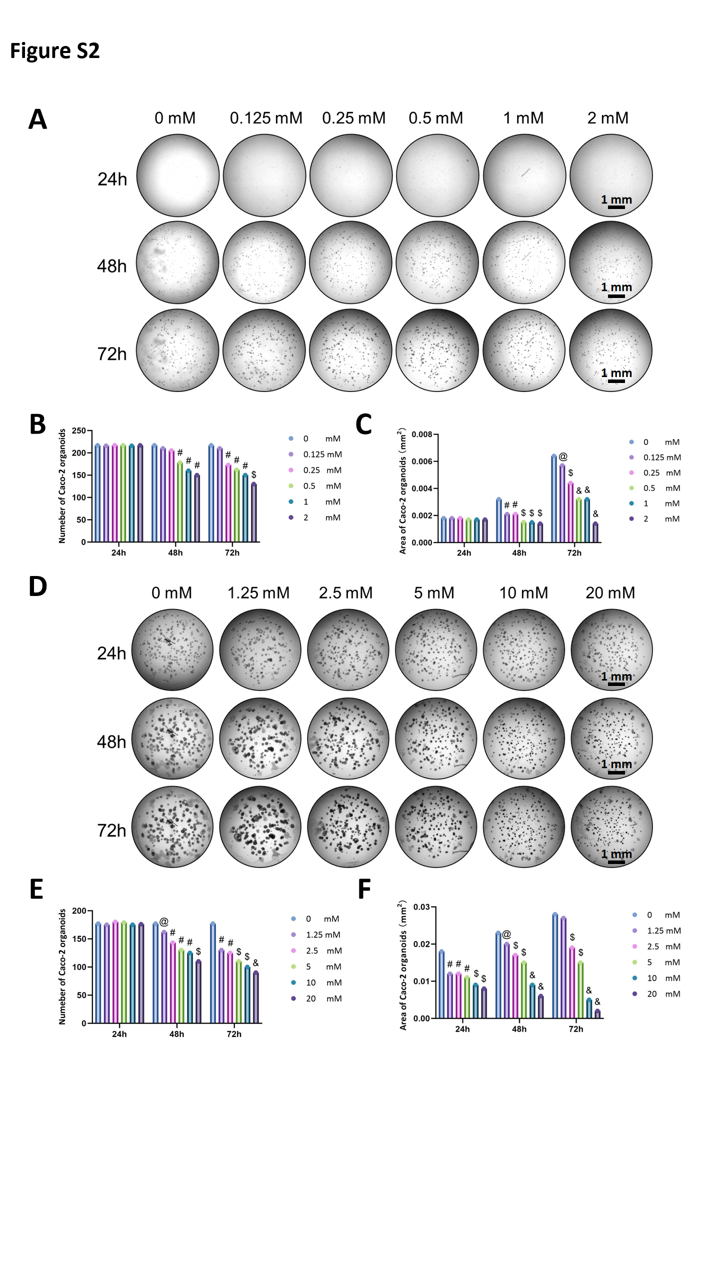
**

**Figure S2. Dose screening of butyrate effects on Caco-2 organoids.** NaB was administered in two different phases. **(A)** Various doses of NaB were loaded at the early formation stage of Caco-2 organoids (Bar=1mm). **(B)** Statistical results for the quantity of Caco-2 organoids at the early stage of growth. **(C)** Quantitative analysis of the area of Caco-2 organoids at the early growth stage. **(D)** After the mature of Caco-2 organoids, NaB with a range of 1.25 mM to 20 mM apparently inhibited the further growth of mature Caco-2 organoids (Bar=1mm). **(E)** Quantitative analysis for the amount of mature Caco-2 organoids at different times after NaB treatment. **(F)** Quantitative analysis for the area of mature Caco-2 organoids after NaB treatment. @: *: *P*<0.05, #: **: *P*<0.01, $: ***: *P*<0.001, &: ****: P <0.0001; n.s: not significant.

**
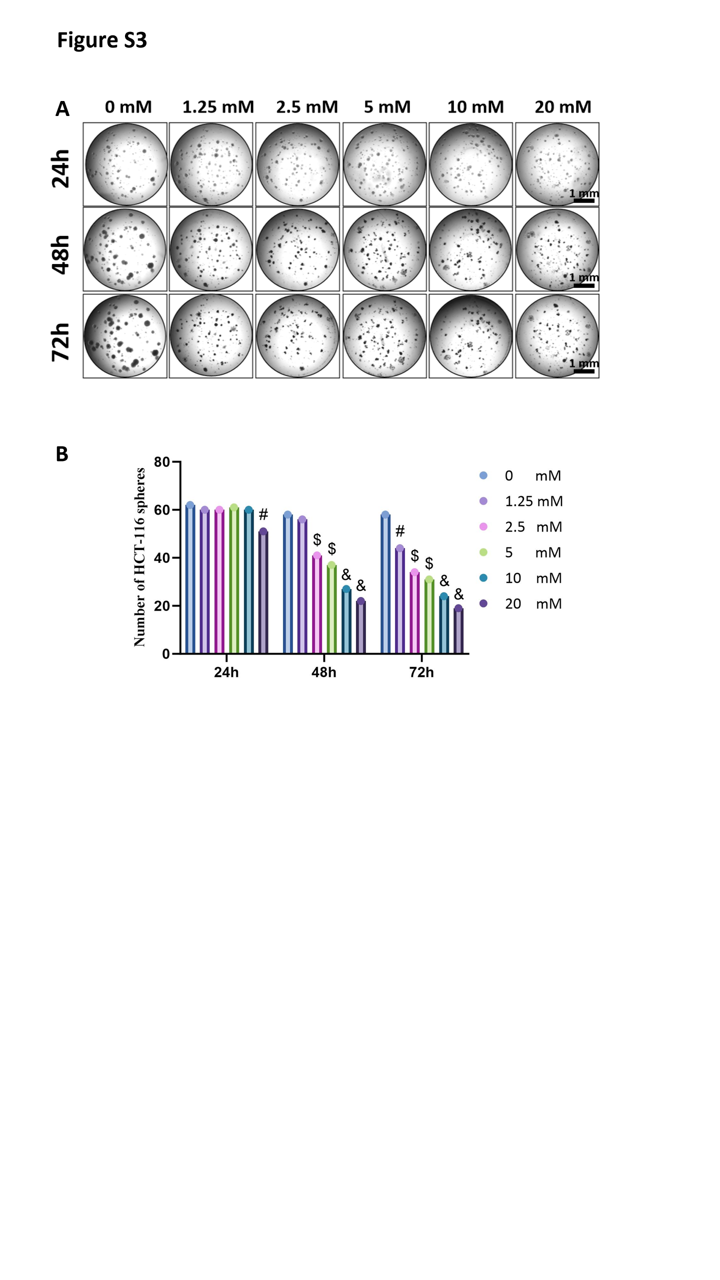
**

**Figure S3. Butyrate suppresses the expansion of CRC cell line derived spheres in a time- and dose-dependent manner.** (A) NaB inhibited the growth of CRC spheres derived from HCT-116 cells (Bar=1mm). (B) NaB significantly decreased the number of HCT-116 spheres in a dose- and time- dependent pattern. #: **: *P*<0.01, $: ***: *P*<0.001, &: ****: P <0.0001; n.s: not significant.


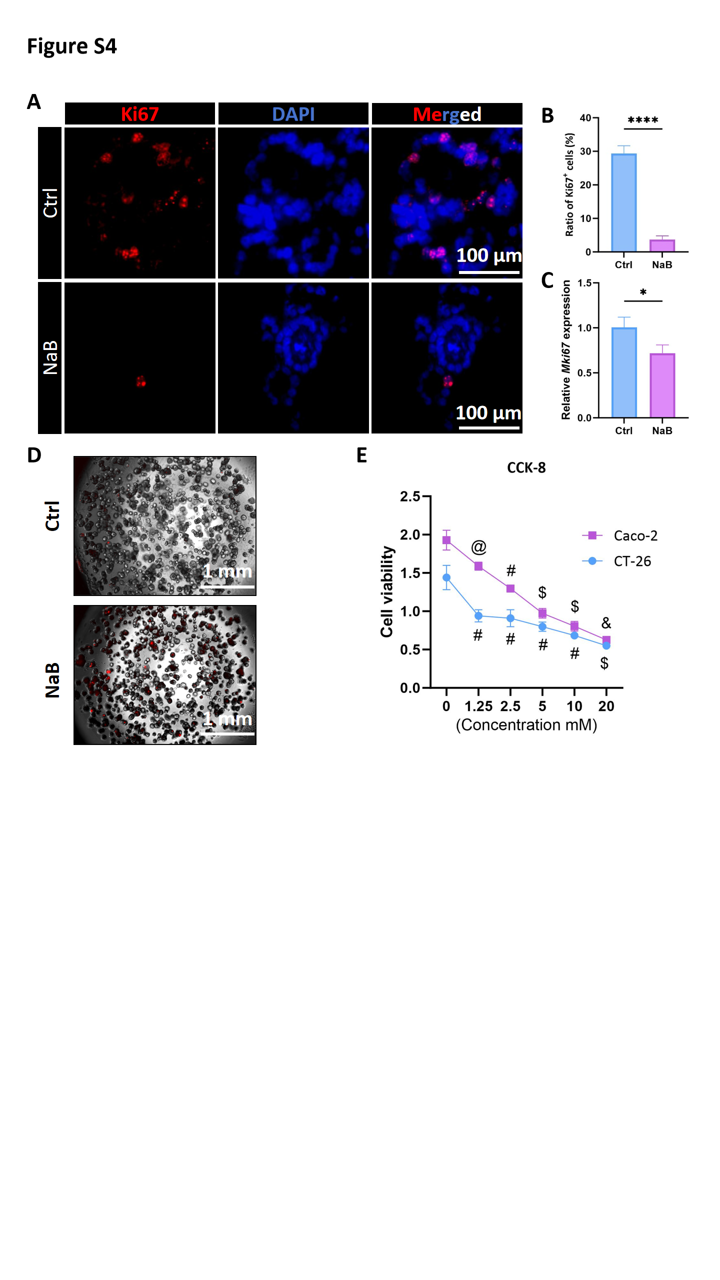


**Figure S4. Butyrate suppresses cell proliferation and induces cell death in Caco-2 organoids. (A)** NaB treatment also decreased the number of Ki67^+^ epithelial cells in Caco-2 organoids (Bar=100µm). **(B)** Statistical analysis for the ratio of Ki67^+^ positive cells in Caco-2 organoids. **(C)** Relative gene expression of *Mki67* in Caco-2 organoids with or without NaB treatment. **(D)** PI staining demonstrated that NaB treatment significantly induced cell death in Caco-2 organoids. **(E)** NaB also dramatically reduced cell viability of Caco-2 and CT-26 cells in 2D culture. @: *: *P*<0.05, #: **: *P*<0.01, $: ***: *P*<0.001, &: ****: P <0.0001; n.s: not significant.

**
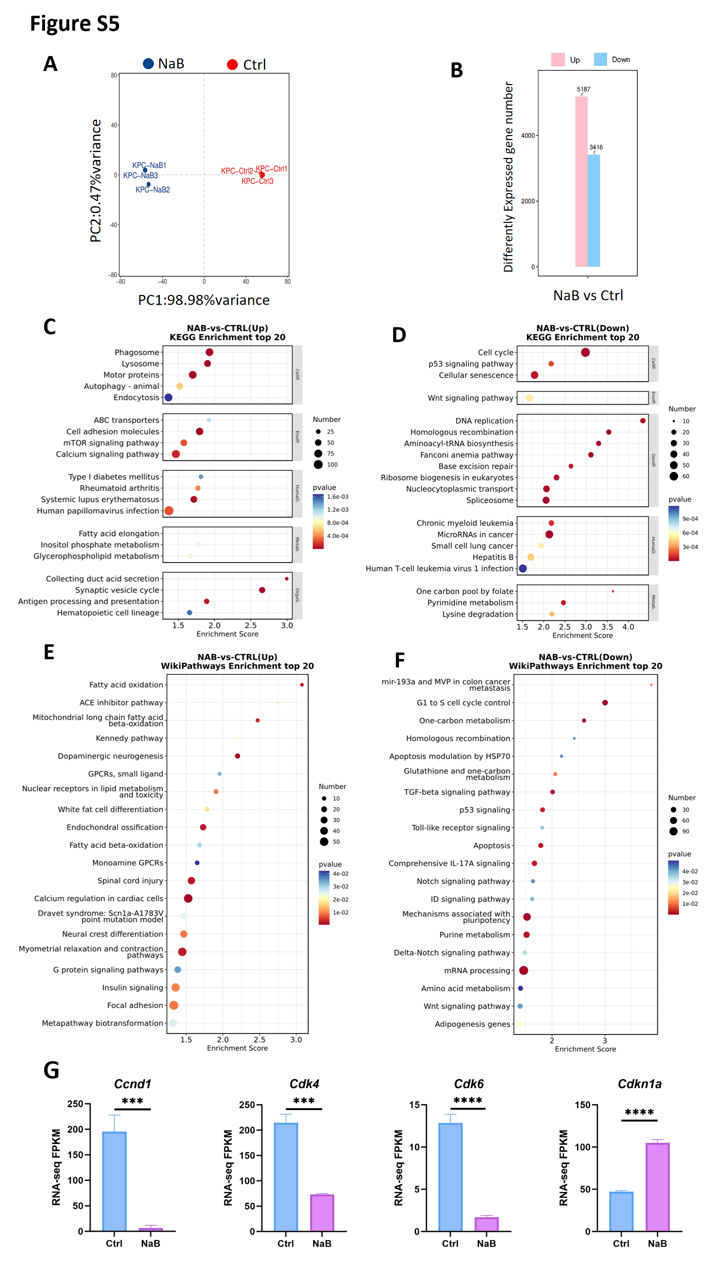
**

**Figure S5. Butyrate suppresses cell cycle related proteins expression in KPC organoids (A)** PCA plot showed distinct transcriptomes between control and NaB-treated KPC organoids (n=3). **(B)** Summary of upregulated and downregulated differentially expressed genes (DEGs). **(C, D)** KEGG pathway analysis revealed the key signaling pathways upregulated and downregulated following sodium butyrate (NaB) treatment. **(E, F)** Wiki pathway analysis revealed the key signaling pathways upregulated and downregulated following sodium butyrate (NaB) treatment.. **(G)** RNA-Seq data revealed significantly reduced FPKM values of cell cycle promoters (*Ccnd1*, *Cdk4*, and *Cdk6*) and increased level of cell cycle inhibitor gene (*Cdkn1a*). ***: *P*<0.001, ****: *P* <0.0001.

**
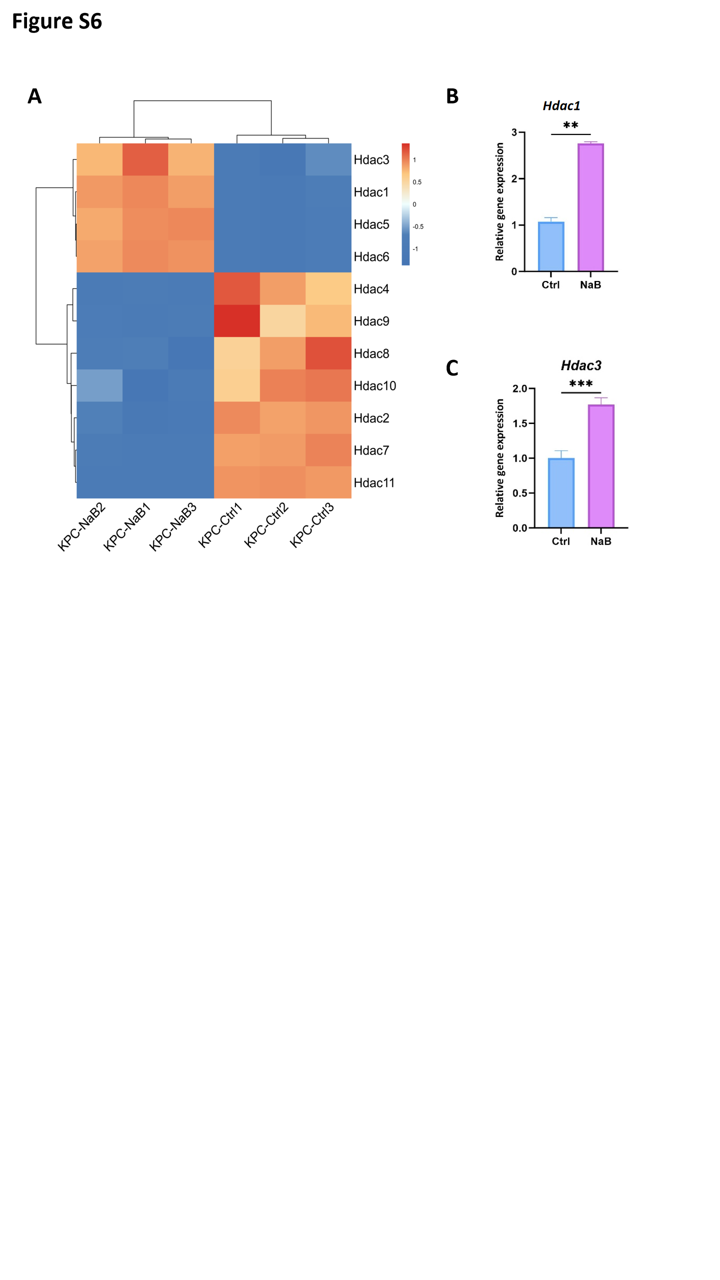
**

**Figure S6.** **Butyrate affects HDACs expression in KPC organoids** **(A)** Heatmap of HDAC family expression profile using RNA-Seq data. **(B,C)** qRT-PCR validation for the gene expression level of *Hdac1* and *Hdac3* in KPC organoids after the administration of NaB. **: *P*<0.01, ***: *P*<0.001.

**
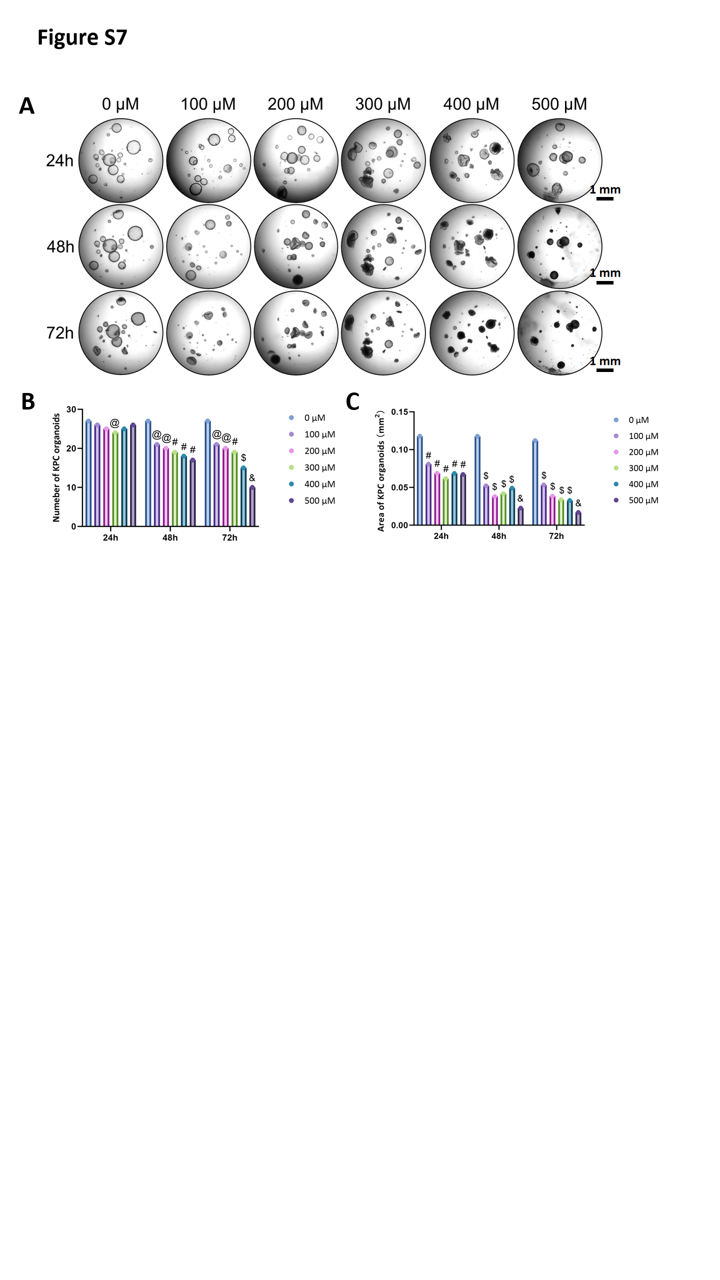
**

**Figure S7. Screening for doses of HDAC2 inhibitor (HDAC2i) on KPC organoids. (A)** Mature KPC organoids were treated with the increasing concentrations of HDAC2i from 100 μM to 500 μM (Bar=1mm). KPC organoids were monitored at 24 h, 48 h, and 72 h after HDAC2i treatment. **(B)** Statistical results for the number of mature KPC organoids following HDAC2i treatment. **(C)** Quantitative analysis for the area of mature KPC organoids after the administration of HDAC2i. @: *: *P*<0.05, #: **: *P*<0.01, $: ***: *P*<0.001, &: ****: P <0.0001; n.s: not significant.

**Table S1. Primer sequences used for qPCR examination**

| **Gene** | **Forward primer** | **Reverse primer** |
| --- | --- | --- |
| hu *Actb* | CATGTACGTTGCTATCCAGGC | CTCCTTAATGTCACGCACGAT |
| hu *Ocln* | GACTTCAGGCAGCCTCGTTAC | GCCAGTTGTGTAGTCTGTCTCA |
| hu *Tjp1* | ACCAGTAAGTCGTCCTGATCC | TCGGCCAAATCTTCTCACTCC |
| ms *Actb* | GTGACGTTGACATCCGTAAAGA | GCCGGACTCATCGTACTCC |
| ms *HDAC1* | AGTCTGTTACTACTACGACGGG | TGAGCAGCAAATTGTGAGTCAT |
| ms *HDAC10* | ACAGCCACTCGACTGCTCT | GATGCCTCACAAGCTGACAAA |
| ms *HDAC11* | GTGTACTCACCACGTTACAACA | GCTCGTTGAGATAGCGCCTC |
| ms *HDAC2* | GGAGGAGGCTACACAATCCG | TCTGGAGTGTTCTGGTTTGTCA |
| ms *HDAC5* | AGCACCGAGGTAAAGCTGAG | GCTGTGGGAGGGAATGGTT |
| ms *HDAC6* | TCCACCGGCCAAGATTCTTC | CAGCACACTTCTTTCCACCAC |
| ms *Mki67* | ATCATTGACCGCTCCTTTAGGT | GCTCGCCTTGATGGTTCCT |
| ms *Ocln* | TCCGGCCGCCAAGGTTC | CATAGCCTCTGTCCCAAGCAA |
| ms *Tjp1* | GCCTTGGCCTAGCATACACA | GGTAAGGCATTCCTGCTGGT |

**Table S2. Information of antibodies used in this study**

| **Antibody** | **Provider** | **Catalog number** | **Host** | **Dilution rate** |
| --- | --- | --- | --- | --- |
| Bax | Proteintech | 50599-2-Ig | Rabbit | 1:400 |
| CDK4 | Cell Signaling | 12790T | Rabbit | 1:500 |
| CDK6 | Cell Signaling | 3136T | Mouse | 1:500 |
| CyclinD1 | Cell Signaling | 2978T | Rabbit | 1:500 |
| HDAC2 | Proteintech | 12922-3-AP | Rabbit | 1:300 |
| Ki67 | Abcam | ab16667 | Rabbit | 1:400 |
| p21 | Cell Signaling | 2947T | Rabbit | 1:500 |
| ZO-1 | Proteintech | 21773-1-AP | Rabbit | 1:200 |
